# Age-dependent decline in learning and memory performances of WAG/Rij rat model of absence epilepsy

**DOI:** 10.1186/1744-9081-8-51

**Published:** 2012-09-22

**Authors:** Ayşe Karson, Tijen Utkan, Fuat Balcı, Feyza Arıcıoğlu, Nurbay Ateş

**Affiliations:** 1 Medical School, Department of Physiology, Kocaeli University, Umuttepe, Kocaeli, 41380, Turkey; 2 Medical School, Department of Pharmacology, Kocaeli University, Kocaeli, Turkey; 3 Department of Psychology, Koç University, Istanbul, Turkey; 4 Faculty of Pharmacy, Department of Pharmacology, Marmara University, Istanbul, Turkey

**Keywords:** Anxiety, Learning, Locomotor Activity, Memory, Rats, WAG/Rij, Wistar

## Abstract

Recent clinical studies revealed emotional and cognitive impairments associated with absence epilepsy. Preclinical research with genetic models of absence epilepsy however have primarily focused on dysfunctional emotional processes and paid relatively less attention to cognitive impairment. In order to bridge this gap, we investigated age-dependent changes in learning and memory performance, anxiety-like behavior, and locomotor activity of WAG/Rij rats (a valid model of generalized absence epilepsy) using passive avoidance, Morris water maze, elevated plus maze, and locomotor activity cage. We tested 5 month-old and 13 month-old WAG/Rij rats and compared their performance to age-matched Wistar rats. Results revealed a decline in emotional and spatial memory of WAG/Rij rats compared to age-matched Wistar rats only at 13 months of age. Importantly, there were no significant differences between WAG/Rij and Wistar rats in terms of anxiety-like behavior and locomotor activity at either age. Results pointed at age-dependent learning and memory deficits in the WAG/Rij rat model of absence epilepsy.

## Introduction

Growing number of studies started to indicate that epilepsy is not restricted to recurrent seizures and emphasize that neuropsychological symptoms are part of its clinical profile [1,2]. Furthermore, emotional and cognitive dysfunction impacts academic performance and social life not only in drug-resistant epilepsy but also benign epileptic syndromes including absence epilepsy, a neurological disorder that lacks structural deficits and responds well to treatment [2-7]. In addition to neuropsychiatric comorbidities, recent studies investigated the time course of neurocognitive impairments in epileptic patients [8-12]. For instance, Hermann et al. reported progressive cognitive abnormalities in temporal lobe epilepsy patients over a 4-year period compared to age- and sex-matched healthy controls [9]. In another study, Hommet et al. compared adolescents and young adults in complete recovery from benign childhood epilepsy with centrotemporal spikes (BECTS) and childhood absence epilepsy (CAE) [8]. They found that patients with CAE showed decline in intellectual performance. Furthermore, brain-imaging studies demonstrated that the brain regions implicated in behavior, cognition, and language had different developmental characteristics in CAE [11,12]. These studies overall emphasize the importance of studying age-related aspects of epilepsy for a better understanding of its multi-faced nature and the development of effective tools for tracking and preventing its progression.

In order to elucidate the relation between behavioral/cognitive impairment and epilepsy, research studies have often used models of acquired epilepsy such as kindling or status epilepticus [13-18]. These models, in which limbic structures are affected, are characterized by convulsive seizures. On the other hand, research on genetic models of absence epilepsy has primarily focused on emotional dysfunction and paid relatively less attention to cognitive impairment [19-22]. Moreover, although genetic models are particularly well-suited for detecting and tracking developmental dynamics, age-related behavioral and cognitive alterations in these models remain unexplored.

Wistar-Albino-Glaxo from Rijswijk (WAG/Rij) rats, a strain of Wistar origin*,* is a widely used, valid genetic model of generalized absence epilepsy [23]. Electrophysiologically and behaviorally well-defined absence seizures occur in every member of WAG/Rij rats [23]. Spike-wave complexes (SWD) begin to emerge on the epidural records of two month-old WAG/Rij rats and their frequency and duration increase with age [23]. SWD activity has been suggested to be closely related to depression-like symptoms observed in this model [19,24]. Unlike the depression-like behavior however, the evaluation of the absence epilepsy models in terms of anxiety-like behaviors has not revealed consistent strain differences [25].

The well-documented impact of epilepsy and depression on learning and memory processes, and the existence of relatively few cognitive characterizations of animal models of absence epilepsy in the literature motivated our study. We examined learning and memory performance of 5 months and 13 months old WAG/Rij rats and age-matched Wistar controls in passive avoidance and Morris water maze, two tasks that are well-characterized in terms of their behavioral, neuroanatomical, and neurochemical bases. We further evaluated the locomotor activity and anxiety-like behavior of 5 m and 13 m WAG/Rij and age-matched Wistar rats using locomotor activity cage and elevated plus maze, respectively. Results revealed age-dependent differences between WAG/Rij and Wistar rats in emotional and spatial memory in the absence of clear differences in terms of anxiety-like behavior and locomotor activity.

## Materials and methods

### Animals

Subjects were 4–6 months old (5 m) and 12–14 months old (13 m), male WAG/Rij and Wistar albino rats. Animals were maintained under standard laboratory conditions on a 12/12-h light/dark cycle (lights on at 7:00 AM). Tap water and food pellets were provided *ad libitum*. Around half of the rats were tested on all behavioral tests on different days. Sixty four rats were tested in locomotor activity cage, 43 rats in elevated plus maze, 71 rats in passive avoidance, and 66 rats in Morris water maze. All experiments were conducted between 09:00 AM and 12:00 PM. All procedures were conducted in accordance with the Regulation of Animal Research Ethics Committee in Turkey (July 6, 2006, Number 26220). Ethical approval was granted by the Kocaeli University Animal Research Ethics Committee (Project number: HADYEK 28, Kocaeli, Turkey).

### Procedure

#### Locomotor activity

Locomotor activity was assessed using an animal activity monitoring system (Commat Ltd., Turkey) composed of a Plexiglas chamber connected to a computer and monitored by an open field activity software. The Plexiglas chamber (40 cm × 40 cm × 35 cm) was equipped with pairs of infrared photo beams and detectors that were mounted horizontally every 2.5 cm and vertically every 4.5 cm. Interruptions of photocell beams were detected and recorded by the software. Total locomotor activity was expressed as the sum of stereotypic, ambulatory, and vertical activity. Activity was monitored continuously for 10 minutes following acclimatization to the test room illuminated with two 36- W overhead fluorescent bulbs (light intensity 190–220 lx in center of the room and 18–45 lx on the cage floors) for a period of one hour.

#### Elevated plus maze

Anxiety-like behavior was evaluated in the elevated plus maze apparatus. Maze was made of wood and consisted of two open arms (50 cm × 10 cm) and two closed arms (50 cm × 10 cm, surrounded by a 40-cm high black wall) connected by a central square (10 cm × 10 cm). Maze was elevated to a height of 50 cm. Rats were placed individually in the central square facing a closed arm and allowed to explore the maze freely for 5 minutes. A rat was considered to have entered an arm when all four limbs were inside the arm. The apparatus was cleaned with ethanol solution after each test. The time spent in closed arms and open/closed arm entries were recorded*.*

#### Passive avoidance

In passive avoidance test, animals learn to inhibit a previously exerted behavior (i.e., moving from the light chamber to the dark chamber). Rats were tested in a step-through type passive avoidance apparatus (Ugo Basile model 7551, Italy). The apparatus (measuring 22 cm × 21 cm × 22 cm) consisted of a light and a dark compartment separated by a guillotine door.

1. *Pre-acquisition trial:* On day one (training trial), rats were placed individually in the light compartment and allowed to explore this compartment. The door between the two compartments was opened after 30 s. During this stage, the animal could freely move into the dark compartment.

2. *Acquisition trial:* The acquisition trial was conducted 15 min after the pre-acquisition trial. Rats were placed in the light compartment. Following a 30 second-long adaptation period, the door separating two compartments was opened. After the rat completely entered the dark compartment (four paws in), the door automatically closed, and an electric foot-shock (0.5 mA) was delivered for 3 seconds via the grid floor. Animals were then removed from the dark compartment and returned to their home cages. The time taken to enter the dark compartment was recorded. Any animal failing to cross from the light to the dark compartment within 300 s was discarded from the experiment. Between each training session, both compartments were cleaned to remove olfactory cues.

3. *Retention trial:* Memory was evaluated 24 h post-training by returning the animals to the light compartment and recording their latency to enter the dark compartment (four paws in). No foot-shock was applied in this trial. If a rat did not enter the dark compartment within 300 s, it was returned to its cage and a maximum latency of 300 s was recorded for that rat. The latency to enter the dark compartment served as a measure of retention performance of step-through avoidance responses.

#### Morris water maze test

The water maze was a circular pool (150 cm in diameter) filled with water (25°C). Small black plastic pieces were placed in the pool in order to make the platform invisible [26]. The pool was located in a dimly lit soundproof test room with a number of extra-maze visual cues.

Maze was divided into four quadrants and three equally spaced points around the edge of the pool were used as starting positions. The order of the release positions was varied throughout the experiment. An escape platform (10 cm diameter) was located in one of the quadrants 1 cm below the water surface during acquisition sessions. Rats were trained in the Morris water maze for five daily sessions (3 trials per session). Five consecutive daily sessions were all performed between 9.00-12.00 h.

For each acquisition trial, a rat was placed in the pool from one of the three locations (randomly chosen) with its head facing toward the wall of the pool. A trial was initiated with the release of the rat in the pool. After the rat had found and climbed onto the platform, the trial was terminated and the mean escape latency was recorded. The maximum trial length was 60 s. If a rat had not climbed onto the platform within 60 s, the trial was terminated and the experimenter guided the rat by hand to the platform. In these cases, an escape latency of 60 s was recorded.

The inter-trial interval was 30 s. During the inter-trial interval the rat was left on the escape platform before starting the next trial. Following the inter-trial interval, the rat was placed in the pool again from a different location and upon its release the next trial began. At the end of each session, the rat was returned to its cage. The escape latency typically declines during acquisition as the animal learns the location of the hidden platform.

Twenty-four hours after the final acquisition session, a ‘probe trial’ was used to assess rats’ retention of the location of the hidden platform. During this trial, the platform was removed from the maze and the rat was allowed to search the pool for 60 s. During this period, animals are expected to spend relatively more time searching the quadrant that previously contained the hidden platform compared to other three quadrants.

### Data analysis

Data from the locomotor activity cage, elevated plus maze, passive avoidance, and probe trials of Morris water maze (separately for two different age groups) were analyzed by one-way ANOVA. Turkey’s HSD was used for post-hoc analysis of significant overall differences. Data from the acquisition trials of Morris water maze were analyzed by a two-way mixed design ANOVA (strain x test day) separately for two different age groups. Data are depicted graphically in terms of average ± SEM. An alpha level of .05 was chosen for statistical significance.

## Results

### Locomotor activity

**Figure 1:**
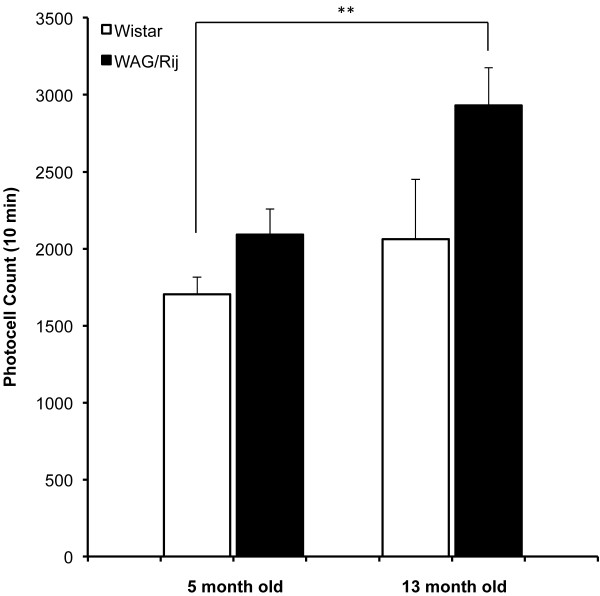
**Total locomotor activity of WAG/Rij and Wistar rats at 5 and 13 months of age.** ** indicates *p* < .01.

Figure 1 shows the total locomotor activity of 5 m and 13 m WAG/Rij and Wistar rats. There was a significant overall difference between the four groups in terms of total locomotor activity, *F*(3,60) = 4.96, *p* < .01. Post-hoc analysis revealed a significant difference only between 5 month-old Wistar and 13 month-old WAG/Rij rats (*p* < .01). There were no significant differences between WAG/Rij and age-matched Wistar rats either at 5 months (*p* = .71) or 13 months of age (*p* = .07).

### Elevated plus maze

**Figure 2:**
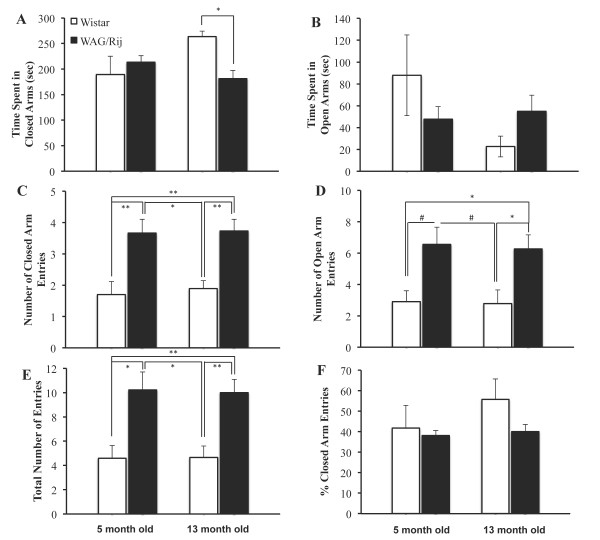
**A) Time spent in closed arms, B) Time spent in open arms, C) number of closed arm entries, D) number of open arm entries, E) total number of arm entries, and F) percentage of closed arm entries in WAG/Rij and Wistar rats at 5 and 13 months of age.** * indicates *p* < .05, ** indicates *p* < .01.

Figure 2 shows the performance in elevated plus maze separately for different behavioral indices. Time spent in closed arms differed significantly between the four groups, *F*(3,39) = 2.89, *p* < .05 (Figure 2A). Post-hoc analysis revealed that WAG/Rij rats spent less time in closed arms than age-matched Wistar rats only at 13 months of age (*p* < .05) and not at 5 months of age (*p* = .88). There was no overall significant differences between the four groups in terms of time spent in open arms, *F*(3,39) = 1.46, *p* = .24 (Figure 2B) or the ratio of time spent in open arms to time spent in open or closed arms, *F*(3,39) = 1.44, *p* = .25.

There were significant overall differences between the four groups in terms of the number of closed arm entries, *F*(3,39) = 7.98, *p* < .001 (Figure 2C), open arm entries, *F*(3,39) = 4.83, *p* < .01 (Figure 2D), and total number of arm entries, *F*(3,39) = 7.27, *p* < .001 (Figure 2E). Post-hoc analysis revealed a significantly higher number of open arm entries of 13 month-old WAG/Rij rats compared to 5 and 13 month-old Wistar (both *p*s < .05). Post-hoc analysis also revealed almost significantly higher number of open arm entries of 5 month-old WAG/Rij rats compared to 5 month-old Wistar rats (*p* = .057) and 13 month Wistar rats (*p* = .054). 5 and 13 month-old WAG/Rij rats exhibited higher number of closed arm and total arm entries than 5 and 13 month-old Wistar rats (all *p*s < .05). There were no significant differences between the four groups in terms of the ratio of open arm entries to the number of entries to open or closed arms, *F*(3,39) = 1.12, *p* < .35 (Figure 2F).

### Passive avoidance

**Figure 3:**
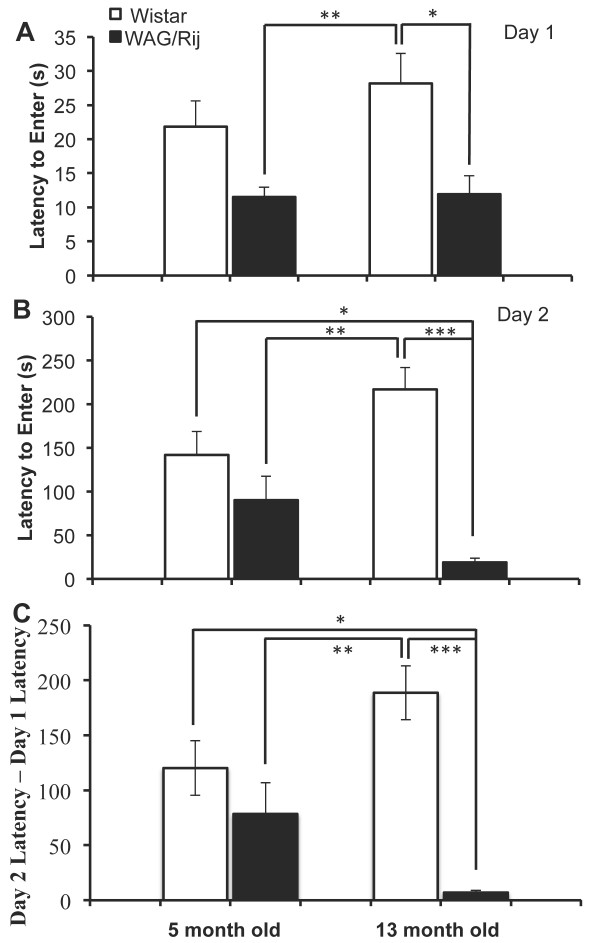
**Latency to enter the dark compartment in the passive avoidance test on A) Day 1 (prior to acquisition) and B) Day 2 (24 hours after acquisiton). C)** The difference in latency to enter the dark compartment between Day 2 and Day 1 [Day 2 Latency – Day 1 Latency]. * indicates *p* < .05, ** indicates *p* < .01, *** indicates *p* < .001.

*Day 1.* There was a significant overall difference between the four groups in terms of time they took to enter the dark compartment, *F*(3,67) = 4.87, *p* < .01 (Figure 3A). Post-hoc analysis revealed that 5 and 13 month-old WAG/Rij rats entered the dark compartment earlier than 13 month-old Wistar rats (both *p*s < .05). There was no significant difference between WAG/Rij and Wistar rats at 5 months of age (*p* = .22).

*Day 2.* There was a significant overall difference between the four groups in terms of time they took to enter the dark compartment, *F*(3,67) = 10.62, *p* < .001 (Figure 3B). Post-hoc analysis revealed that 13 month-old WAG/Rij rats entered the dark compartment earlier than both 5 and 13 month-old Wistar rats (both *p*s < .05) and that 5 month-old WAG/Rij rats entered the dark compartment earlier than 13 month-old Wistar rats (*p* < .01). There was no difference between WAG/Rij and age-matched Wistar rats at 5 months of age (*p* = .48).

*Day 2-Day 1:* The same analyses were conducted on the difference between latency to enter the dark compartment on Day 2 and latency to enter the dark compartment on Day 1 [Latency(Day 2)-Latency(Day 1)]. This analysis showed an overall significant difference between the four groups, *F*(3,67) = 9.40, *p* < .001 (Figure 3C). Post-hoc analysis revealed a smaller difference score for 13 month-old WAG/Rij rats compared to both 5 and 13 month-old Wistar rats (both *p*s < .05) and for 5 month-old WAG/Rij rat compared to 13 month-old Wistar rats (*p* < .01). There was no significant difference between WAG/Rij and age-matched Wistar rats at 5 months of age (*p* = .63).

### Morris water maze

**Figure 4:**
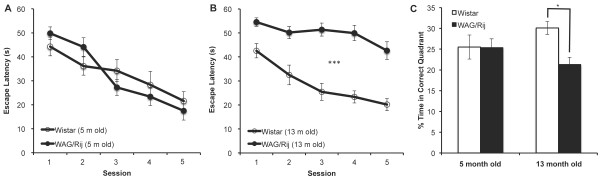
**Escape latency separately for A) 5 month old and B) 13 month old Wistar and WAG/Rij rats.C)** Percentage of time spent in correct quadrant. * indicates *p* < .05, *** indicates *p* < .001.

Figure 4A &4B shows the escape latency as a function of training sessions separately for four different groups (2 strain × 2 age). At 5 months of age, there was no significant difference between WAG/Rij and Wistar rats in terms of escape latency, *F*(1,29) = .02, *p* = .90. As expec-ted, training significantly decreased the escape latency, *F*(4,116) = 24.30, *p* < .001. There was no strain by training interaction, *F*(4,116) = 2.29, *p* = .06. At 13 months of age, WAG/Rij rats were slower at finding the platform compared to age-matched Wistar rats, *F*(1,33) = 61.03, *p* < .001. As in the case of 5 m groups, training significantly decreased the escape latency, *F*(4,132) = 10.23, *p* < .001. There was no strain by training interaction, *F*(4,132) = 2.4, *p* = .053.

Since we could not evaluate the swimming speed and had not tested rats on a cued platform session, we repeated the analysis after normalizing escape latencies on each session by the corresponding escape latencies observed on the first session. Results of this analysis corroborated our findings based on absolute escape latencies. There was no difference between WAG/Rij and Wistar rats at 5 months of age, *F*(1,29) = 2.50, *p* = .13. There was a significant effect of training on normalized escape latencies, *F*(3,87) = 14.68, *p* < .001 and there was no significant strain by training interaction, *F*(3,87) = 2.22, *p* = .09 at 5 months of age. At 13 months of age, WAG/Rij rats exhibited significantly longer normalized escape latencies than age-matched Wistar rats, *F*(1,33) = 6.90, *p* < .05. Training significantly decreased the normalized escape latencies, *F*(3,99) = 4.37, *p* < .01 at 13 months of age. There was no significant strain by training interaction, *F*(3,99) = 1.04, *p* = .38 at this age group.

There was an overall significant difference between the four groups in terms of the percentage of time spent in the correct quadrant, *F*(3,62) = 3.19, *p* < .05 (Figure 4C). Post-hoc analysis showed that WAG/Rij rats spent less time in the correct quadrant compared to Wistar rats only at 13 months of age (*p* < .05) and not at 5 months of age (*p* = .99).

## Discussion

WAG/Rij rats exhibited worse emotional and spatial memory performances in passive avoidance and Morris water maze tests compared to their aged-matched Wistar controls at 13 months but not at 5 months of age. Importantly, these cognitive deficits were observed in the absence of clear differences in anxiety-like behavior and locomotor activity. For instance, WAG/Rij rats did not exhibit different levels of anxiety-like behavior (i.e., time spent in open arms or the ratio of time spent in open arms to time spent in open or closed arms) in the elevated plus maze or different levels of locomotor activity in the activity cage compared to their age-matched Wistar controls. These results overall suggested an age-dependent decline in the learning and memory performances of WAG/Rij rat model of absence epilepsy (compared to Wistar controls) that cannot be accounted for by differential levels of anxiety and locomotor activity.

The idiopathic generalized epilepsy, characterized with typical absence seizures, has been assumed to be a type of benign epilepsies. However, recent studies led to a change in this assumption except for the responsiveness of these conditions to anti-epileptic treatment [11,12,27]. Clinical studies carried out with absence epileptic patients indicate presence of cognitive impairments including general cognitive decline [28], visiospatial dysfunction [28,29], linguistic problems [26], non-verbal and short-term verbal memory impairment [26,29] as well as attentional, emotional and behavioral alterations [9,29-32]. Several of these studies have been conducted with non-medicated patients with newly emerging seizures [9,29]. In humans, several variables including medical treatment and social factors make it difficult to detect the onset and track the progression of cognitive impairments [33].

In animal models of absence epilepsy, cognitive functions have received surprisingly little attention. In one of these studies, WAG/Rij rats were tested on the hole board and radial arm maze, and exhibited a partial decline of the reference memory without the working memory impairment [34]. Bazyan et al. ([2000]) also showed that WAG/Rij rats performed worse than controls in the passive avoidance test [35]. In the same study, although WAG/Rij rats performed better than controls early in training in the active avoidance test, their performance worsened during latter trials [35]. These results emphasize the task-specificity of memory performance in the absence epilepsy models. None of these studies however, have addressed the possible age-dependent dynamics in memory performance.

Our study clearly demonstrates that memory impairment became evident in WAG/Rij rats even over the course of a limited age range (i.e., 5 m/young adulthood vs. 13 m/middle-aged). One plausible causal basis of this age-dependent decline in memory performance of WAG/Rij rats in the light of previous studies is their differential spike-wave discharge (SWD) activity compared to age-matched Wistar rats. For instance, previous studies showed an age-dependent increase in the frequency of absence seizures in WAG/Rij rats; SWD activity starts between 2–3 months of age and reaches hundreds a day by 12 months of age, the age group tested in this study [23,36]. SWD activity does not only appear in these inbred absence epilepsy models but also in g/Cpb rats [37], APO-SUS [38], and Long Evans [39] rats. Importantly, these rat models too exhibit impaired performance on different memory and/or emotional tasks [37,38,40]. SWD activity was further observed in all members of the outbreed Wistar rats within the 15–23 m age range [41] and can be detected even earlier in this strain [42]. Again consistent with SWD-based account, Wistar rats exhibit poor performance in passive avoidance and water maze tests within the older age range characterized by SWD activity [43-45]. Thus, the behavioral results of the current study combined with electrophysiological findings of previous studies point at a possible relation between SWD activity and age-dependent memory impairment of WAG/Rij rats. Note that since we did not record EEG in this study, this relation only remains to be a plausible account that needs to be substantiated by data. Thus, future studies are needed to establish the relation between SWD activity and memory performance by recording EEG from rats tested in learning and memory assays.

Despite these previous findings that suggest a potential relationship between SWD activity and cognitive dysfunction, results obtained from GAERS (genetic absence epilepsy rats from Strasbourg) appear to conflict this relation. The SWD activity emerges earlier, occurs more frequent and with longer duration in GAERS, another genetic rat model of absence epilepsy [23]. Thus, under an SWD-based account GAERS would be predicted to exhibit poorer memory performance earlier than observed here. Contrary to this prediction, GAERS rats performed better than controls in the two-way active avoidance test [46]. The disparity between the effect of brain lesions on two-way active avoidance and other memory task performances suggest that this contradiction might be task-specific.

For instance, hippocampal, septohippocampal or reticular thalamic nucleus lesions, which had detrimental effects on performances in many memory tasks, have been shown to enhance two-way active avoidance performance [47-51]. Based on this disparity, it has been suggested that two-way active avoidance performance entailed complex aversive learning, and that it could be facilitated by reduced emotionality, decreased cholinergic innervation, and increased behavioral disinhibition [50,52]. The study of GAERS rats in other memory tests would help elucidate whether their enhanced performance in two-way active avoidance is task specific or not.

A neuroanatomical perspective would contribute to the understanding of the dynamics that underlie our behavioral results. Limbic structures including amygdala, hippocampus, and parahippocampal cortical areas are particularly implicated for the performance on the two memory tasks used in this study [53-56]. These structures were evaluated previously by electrophysiological, molecular, and functional methods in the absence epileptic rats. Electrophysiological studies have shown that limbic areas were silent during thalamocortical SWD activity [57,58]. On the other hand, other studies reported the presence of the metabolic and molecular changes [59-62]. Support for functional changes in the limbic structures of absence epileptic rats originate from kindling studies. Kindling, which is a model of epileptogenesis for acquired epilepsies, shares some of the similar mechanisms with long-term potentiation (LTP) [57]. In fact, it is considered to be a pathological form of neuroplasticity [63,64]. Development of kindling has been shown to be more difficult in rats with absence epilepsy and different degrees of resistance to kindling progression have been shown in these animals in amygdala, hippocampus, and perirhinal cortex [65-67]. If resistance to kindling in these rat models indeed generalizes to the formation of LTP in the corresponding limbic structures, this could predict worse learning and memory performance in absence epilepsy rat models.

Finally, the performance of rats in these tests might be altered by other factors such as emotional state, motivation and/or impulsivity. For instance, in a recent study [18] of two models of temporal lobe epilepsy in two different strains, researchers found that lower anxiety scores (as assessed by open arm related measures) in EPM was related to lower performance on the Morris water maze. Although we did not observe any differences between WAG/Rij and Wistar rats in terms of anxiety-like behavior as canonically assessed by open arm related measures, WAG/Rij rats spent less time in closed arms compared to age-matched Wistar rats at 13 months of age. This result might imply weaker safety-seeking tendencies of WAG/Rij rats and might indeed be relevant to their lower performance on passive avoidance and Morris water maze, which are tasks with aversive components. Contrary to these arguments however, van der Staay et al. ([2009]) demonstrated that emotional reactivity as assessed by open field and elevated plus maze activities did not mediate learning and memory performance in tasks with aversive components such as Morris water maze and passive avoidance [68].

In our study, WAG/Rij rats did not exhibit different levels of locomotor activity in the activity cage however they exhibited higher total number of arm entries in EPM at both ages. Although, these differences might be argued to have mediated the performance on passive avoidance and Morris water maze, this reasoning cannot fully account for the age-dependence of poorer performance on these memory tasks. In other words, differences between WAG/Rij and Wistar rats in terms of arm entries was comparable between the two age groups whereas cognitive impairment appeared specifically at 13 months of age. The same argument also holds for the possible relation between number of arm entries and time-spent in closed arms; the between-strain differences for the former measure was not age-dependent whereas age-dependency of between-strain differences held for the latter. Furthermore, although previous studies argued for a relation between higher levels of activity in elevated plus maze and open arm exploration [69], its relation to closed arm exploration has not been discussed.

We did not test rats on a cued-learning trial in order to control for possible factors (other than spatial memory) that might have mediated the observed performances. However, note that analysis of the escape latencies normalized by the escape latency of the first session also revealed similar results. We observed that WAG/Rij rats at 13 months of age jumped of the platform upon being placed on it by the experimenter after the trial cut-off duration. This behavior was not observed with any Wistar rats or 5 months old WAG/Rij rats. Further studies should evaluate the swimming speed and other potentially confounding factors.

In conclusion, this study shows an age-dependent decline in the learning and memory performance of WAG/Rij rats compared to age-matched Wistar rats in the absence of clear differences in anxiety-like behavior and locomotor activity. More detailed studies are needed to understand the electrophysiological correlates of these behavioral alterations as well as the relation between the neuroanatomical and neurochemical components of this disturbance and other emotional/behavioral comorbid situations.

## Competing interests

The authors declare that they have no competing interests.

## Authors’ contributions

AK, TU, FA, and NA designed the experiments. AK and TU conducted the experiments. AK and FB analyzed the data. AK, TU, FB, and NA drafted the manuscript. All authors read and approved the final manuscript.

## References

[B1] Berg AT Epilepsy, cognition, and behavior: the clinical picture Epilepsia 2011 52 Suppl 1 7 12 21214534 10.1111/j.1528-1167.2010.02905.x PMC3057769

[B2] Plioplys S Dunn DW Caplan R 10-year research update review: psychiatric problems in children with epilepsy J Am Acad Child Adolesc Psychiatry 2007 46 1389 1402 10.1097/chi.0b013e31815597fc 18049289

[B3] Baxendale S Heaney D Thompson PJ Duncan JS Cognitive consequences of childhood-onset temporal lobe epilepsy across the adult lifespan Neurology 2010 75 705 711 10.1212/WNL.0b013e3181eee3f0 20733146

[B4] Stretton J Thompson PJ Frontal lobe function in temporal lobe epilepsy Epilepsy Res 2012 98 1 13 10.1016/j.eplepsyres.2011.10.009 22100147 PMC3398387

[B5] Wirrell EC Camfield CS Camfield PR Dooley JM Gordon KE Smith B Long-term psychosocial outcome in typical absence epilepsy. Sometimes a wolf in sheeps’ clothing Arch Pediatr Adolesc Med 1997 151 152 158 10.1001/archpedi.1997.02170390042008 9041870

[B6] Conant LL Wilfong A Inglese C Schwarte A Dysfunction of executive and related processes in childhood absence epilepsy Epilepsy Behav 2010 18 414 423 10.1016/j.yebeh.2010.05.010 20656561

[B7] Chan SC Lee WT Benign epilepsy in children J Formos Med Assoc 2011 110 134 144 10.1016/S0929-6646(11)60023-5 21497276

[B8] Hommet C Billard C Motte J Passage G Perrier D Gillet P Prunier C Toffol B Autret A Cognitive function in adolescents and young adults in complete remission from benign childhood epilepsy with centro-temporal spikes Epileptic Disorders 2001 3 207 216 11844716

[B9] Hermann BP Jones JE Sheth R Koehn M Becker T Fine J Allen CA Seidenberg M Growing up with epilepsy: a two-year investigation of cognitive development in children with new onset epilepsy Epilepsia 2008 49 1847 1858 10.1111/j.1528-1167.2008.01735.x 18785880 PMC2921826

[B10] Helmstaedter C Elger CE Chronic temporal lobe epilepsy: a neurodevelopmental or progressively dementing disease? Brain 2009 132 2822 2830 10.1093/brain/awp182 19635728

[B11] Tosun D Siddarth P Toga AW Hermann B Caplan R Effects of childhood absence epilepsy on associations between regional cortical morphometry and aging and cognitive abilities Hum Brain Mapp 2011 32 580 591 10.1002/hbm.21045 21391248 PMC3058615

[B12] Caplan R Levitt J Siddarth P Wu KN Gurbani S Sankar R Shields WD Frontal and temporal volumes in Childhood Absence Epilepsy Epilepsia 2009 50 2466 2472 10.1111/j.1528-1167.2009.02198.x 19624714

[B13] Depaulis A Helfer V Deransart C Marescaux C Anxiogenic-like consequences in animal models of complex partial seizures Neurosci Biobehav Rev 1997 21 767 774 10.1016/S0149-7634(96)00060-7 9415901

[B14] Kalynchuk LE Long-term amygdala kindling in rats as a model for the study of interictal emotionality in temporal lobe epilepsy Neurosci Biobehav Rev 2000 24 691 704 10.1016/S0149-7634(00)00031-2 10974352

[B15] Hannesson DK Howland J Pollock M Mohapel P Wallace AE Corcoran ME Dorsal hippocampal kindling produces a selective and enduring disruption of hippocampally mediated behavior J Neurosci 2001 21 4443 4450 11404431 10.1523/JNEUROSCI.21-12-04443.2001 PMC6762750

[B16] Erdoğan F Gölgeli A Arman F Ersoy AO The effects of pentylenetetrazole-induced status epilepticus on behavior, emotional memory, and learning in rats Epilepsy Behav 2004 5 388 393 10.1016/j.yebeh.2004.03.001 15145309

[B17] Cardoso A Carvalho LS Lukoyanova EA Lukoyanov NV Effects of repeated electroconvulsive shock seizures and pilocarpine-induced status epilepticus on emotional behavior in the rat Epilepsy Behav 2009 14 293 299 10.1016/j.yebeh.2008.11.004 19068237

[B18] Inostroza M Cid E Brotons-Mas J Gal B Aivar P Uzcategui YG Sandi C Menendez De La Prida L Hippocampal-dependent spatial memory in the water maze is preserved in an experimental model of temporal lobe epilepsy in rats PLoS One 2011 6 e22372 10.1371/journal.pone.0022372 21829459 PMC3144225

[B19] Sarkisova KY Midzianovskaia IS Kulikov MA Depressive-like behavioral alterations and c-fos expression in the dopaminergic brain regions in WAG/Rij rats with genetic absence epilepsy Behav Brain Res 2003 144 211 226 10.1016/S0166-4328(03)00090-1 12946611

[B20] Midzyanovskaya IS Shatskova AB Sarkisova KY van Luijtelaar G Tuomisto L Kuznetsova GD Convulsive and nonconvulsive epilepsy in rats: effects on behavioral response to novelty stress Epilepsy Behav 2005 6 543 551 10.1016/j.yebeh.2005.03.005 15907748

[B21] Jones NC Salzberg MR Kumar G Couper A Morris MJ O’Brien TJ Elevated anxiety and depressive-like behavior in a rat model of genetic generalized epilepsy suggesting common causation Exp Neurol 2008 209 254 260 10.1016/j.expneurol.2007.09.026 18022621

[B22] Sarkisova KY Kuznetsova GD Kulikov MA van Luijtelaar G Spike-wave discharges are necessary for the expression of behavioral depression-like symptoms Epilepsia 2010 51 146 160 10.1111/j.1528-1167.2009.02260.x 19674046

[B23] Coenen AM Van Luijtelaar EL Genetic animal models for absence epilepsy: a review of the WAG/Rij strain of rats Behav Genet 2003 33 635 655 10.1023/A:1026179013847 14574120

[B24] Sarkisova K van Luijtelaar G The WAG/Rij strain: a genetic animal model of absence epilepsy with comorbidity of depressiony Prog Neuropsychopharmacol Biol Psychiatry 2011 35 854 876 10.1016/j.pnpbp.2010.11.010 21093520

[B25] van Luijtelaar G The prevention of behavioral consequences of idiopathic generalized epilepsy: evidence from rodent models Neurosci Lett 2011 497 177 184 10.1016/j.neulet.2011.02.034 21354267

[B26] Pothion S Bizot JC Trovero F Belzung C Strain differences in sucrose preference and in the consequences of unpredictable chronic mild stress Behav Brain Res 2004 155 135 146 10.1016/j.bbr.2004.04.008 15325787

[B27] Sinclair DB Unwala H Absence epilepsy in childhood: electroencephalography (EEG) does not predict outcome J Child Neurol 2007 22 799 802 10.1177/0883073807304198 17715268

[B28] Pavone P Bianchini R Trifiletti RR Incorpora G Pavone A Parano E Neuropsychological assessment in children with absence epilepsy Neurology 2001 56 1047 1051 10.1212/WNL.56.8.1047 11320177

[B29] Bhise VV Burack GD Mandelbaum DE Baseline cognition, behavior, and motor skills in children with new-onset, idiopathic epilepsy Dev Med Child Neurol 2010 52 22 26 10.1111/j.1469-8749.2009.03404.x 19702836

[B30] Caplan R Siddarth P Stahl L Lanphier E Vona P Gurbani S Koh S Sankar R Shields WD Childhood absence epilepsy: behavioral, cognitive, and linguistic comorbidities Epilepsia 2008 49 1838 1846 10.1111/j.1528-1167.2008.01680.x 18557780

[B31] Vega C Vestal M DeSalvo M Berman R Chung M Blumenfeld H Spann MN Differentiation of attention-related problems in childhood absence epilepsy Epilepsy Behav 2010 19 82 85 10.1016/j.yebeh.2010.06.010 20674507 PMC2943027

[B32] Vega C Guo J Killory B Danielson N Vestal M Berman R Martin L Gonzalez JL Blumenfeld H Spann MN Symptoms of anxiety and depression in childhood absence epilepsy Epilepsia 2011 52 e70 e74 10.1111/j.1528-1167.2011.03119.x 21635244 PMC3145036

[B33] Hixson JD Kirsch HE The effects of epilepsy and its treatments on affect and emotion Neurocase 2009 15 206 216 10.1080/13554790802632876 19204849

[B34] van Luijtelaar EL van der Staay FJ Kerbusch JM Spatial memory in rats: a cross validation study Q J Exp Psychol B 1989 41 287 306 2798928

[B35] Bazyan AS Getsova VM Orlova NV Kuznetsova GD, Coenen A, Chepurnov SA, Luijtelaar G Characterization of learning and memory in WAG/Rij rats prone to absence epilepsy The WAG/Rij rat model of absence epilepsy: the Nijmegen– Moscow research. A tribute to five years of co-operation 2000 Nijmegen: Nijmegen University Press 99 104

[B36] Marescaux C Vergnes M Depaulis A Genetic absence epilepsy in rats from Strasbourg–a review J Neural Transm Suppl 1992 35 37 69 1512594 10.1007/978-3-7091-9206-1_4

[B37] Inoue M Peeters BW van Luijtelaar EL Vossen JM Coenen AM Spontaneous occurrence of spike-wave discharges in five inbred strains of rats Physiol Behav 1990 48 199 201 10.1016/0031-9384(90)90285-C 2122483

[B38] Cools AR Dierx J Coenders C Heeren D Ried S Jenks BG Ellenbroek B Apomorphine-susceptible and apomorphine-unsusceptible Wistar rats differ in novelty-induced changes in hippocampal dynorphin B expression and two-way active avoidance: a new key in the search for the role of the hippocampal-accumbens axis Behav Brain Res 1993 55 213 221 10.1016/0166-4328(93)90117-9 8102850

[B39] Shaw FZ Is spontaneous high-voltage rhythmic spike discharge in Long Evans rats an absence-like seizure activity? J Neurophysiol 2004 91 63 77 12826656 10.1152/jn.00487.2003

[B40] Shaw FZ Chuang SH Shieh KR Wang YJ Depression- and anxiety-like behaviors of a rat model with absence epileptic discharges Neuroscience 2009 5 160 382 393 19272419 10.1016/j.neuroscience.2009.02.053

[B41] van Luijtelaar EL Ates N Coenen AM Role of L-type calcium channel modulation in nonconvulsive epilepsy in rats Epilepsia 1995 36 86 92 10.1111/j.1528-1157.1995.tb01671.x 7528137

[B42] Chocholová L Incidence and development of rhythmic episodic activity in the electroencephalogram of a large rat population under chronic conditions Physiol Bohemoslov 1983 32 10 18 6844444

[B43] Jucker M Oettinger R Battig K Age-related changes in working and reference memory performance and locomotor activity in the Wistar rat Behav Neural Biol 1988 50 24 36 10.1016/S0163-1047(88)90744-3 3401195

[B44] Kadar T Silbermann M Brandeis R Levy A Age-related structural changes in the rat hippocampus: correlation with working memory deficiency Brain Res 1990 512 113 120 10.1016/0006-8993(90)91178-J 2337798

[B45] Miettinen R Sirviö J Riekkinen P Sr Laakso MP Riekkinen M Jr Riekkinen P Neocortical, hippocampal and septal parvalbumin- and somatostatin-containing neurons in young and aged rats: correlation with passive avoidance and water maze performance Neuroscience 1993 53 367 378 10.1016/0306-4522(93)90201-P 8098509

[B46] Getova D Bowery NG Spassov V Effects of GABAB receptor antagonists on learning and memory retention in a rat model of absence epilepsy Eur J Pharmacol 1997 320 9 13 10.1016/S0014-2999(96)00877-1 9049596

[B47] Olton DS Isaacson RL Hippocampal lesions and active avoidance Physiol Behav 1968 3 719 724 10.1016/0031-9384(68)90142-X

[B48] Schwartzbaum JS Green RH Beatty WW Thompson JB Acquisition of avoidance behavior following septal lesions in the rat J Comp Physiol Psychol 1967 63 95 104 6029728 10.1037/h0024145

[B49] Comparative effects of septo-hippocampal and caudate lesions on avoidance behavior in rats J Comp Physiol Psychol 1967 64 444 452 6082875 10.1037/h0025195

[B50] Guillazo-Blanch G Nadal R Vale-Martínez A Martí-Nicolovius M Arévalo R Morgado-Bernal I Effects of fimbria lesions on trace two-way active avoidance acquisition and retention in rats Neurobiol Learn Mem 2002 78 406 425 10.1006/nlme.2002.4073 12431426

[B51] Tenas-Huerga N Coll-Andreu M Guillazo-Blanch G Martí-Nicolovius M Morgado-Bernal I Facilitatory effects of thalamic reticular nucleus lesions on two-way active avoidance in rats Exp Brain Res 1998 118 511 516 10.1007/s002210050307 9504846

[B52] Carballo-Márquez A Boadas-Vaello P Villarejo-Rodríguez I Guillazo-Blanch G Martí-Nicolovius M Vale-Martínez A Effects of muscarinic receptor antagonism in the basolateral amygdala on two-way active avoidance Exp Brain Res 2011 209 455 464 10.1007/s00221-011-2576-4 21318348

[B53] Baldi E Ambrogi Lorenzini C Sacchetti B Tassoni G Bucherelli C Effects of coupled perirhinal cortex and medial septal area, fimbria-fornix, entorhinal cortex tetrodotoxin inactivations on passive avoidance consolidation in the rat Neurosci Lett 2000 280 91 94 10.1016/S0304-3940(00)00783-7 10686385

[B54] Burwell RD Saddoris MP Bucci DJ Wiig KA Corticohippocampal contributions to spatial and contextual learning J Neurosci 2004 24 3826 3836 10.1523/JNEUROSCI.0410-04.2004 15084664 PMC6729354

[B55] Wilensky AE Schafe GE LeDoux JE The amygdala modulates memory consolidation of fear-motivated inhibitory avoidance learning but not classical fear conditioning J Neurosci 2000 20 7059 7066 10995852 10.1523/JNEUROSCI.20-18-07059.2000 PMC6772812

[B56] Cimadevilla JM Méndez-López M Méndez M Arias JL Interhippocampal transfer in passive avoidance task modifies metabolic activity in limbic structures Hippocampus 2011 21 48 55 10.1002/hipo.20720 19921701

[B57] Coenen AM Drinkenburg WH Inoue M van Luijtelaar EL Genetic models of absence epilepsy, with emphasis on the WAG/Rij strain of rats Epilepsy Res 1992 12 75 86 10.1016/0920-1211(92)90029-S 1396543

[B58] Vergnes M Marescaux C Depaulis A Mapping of spontaneous spike and wave discharges in Wistar rats with genetic generalized non-convulsive epilepsy Brain Res 1990 523 87 91 10.1016/0006-8993(90)91638-W 2207693

[B59] Lasoń W Przewłocka B Van Luijtelaar G Coenen A Proenkephalin and prodynorphin mRNA level in brain of rats with absence epilepsy Neuropeptides 1994 27 343 347 10.1016/0143-4179(94)90060-4 7898641

[B60] Nehlig A Vergnes M Boyet S Marescaux C Local cerebral glucose utilization in adult and immature GAERS Epilepsy Res 1998 32 206 212 10.1016/S0920-1211(98)00052-7 9761321

[B61] Lakaye B de Borman B Minet A Arckens L Vergnes M Marescaux C Grisar T Increased expression of mRNA encoding ferritin heavy chain in brain structures of a rat model of absence epilepsy Exp Neurol 2000 162 112 120 10.1006/exnr.2000.7303 10716893

[B62] Danış O Demir S Günel A Aker RG Gülçebi M Onat F Ogan A Changes in intracellular protein expression in cortex, thalamus and hippocampus in a genetic rat model of absence epilepsy Brain Res Bull 2011 84 381 388 10.1016/j.brainresbull.2011.02.002 21310218

[B63] McEachern JC Shaw CA The plasticity-pathology continuum: defining a role for the LTP phenomenon J Neurosci Res 1999 58 42 61 10.1002/(SICI)1097-4547(19991001)58:1<42::AID-JNR6>3.0.CO;2-L 10491571

[B64] Meador KJ The basic science of memory as it applies to epilepsy Epilepsia 2007 48 Suppl 9 23 25 18047596 10.1111/j.1528-1167.2007.01396.x

[B65] Eşkazan E Onat FY Aker R Oner G Resistance to propagation of amygdaloid kindling seizures in rats with genetic absence epilepsy Epilepsia 2002 43 1115 1119 10.1046/j.1528-1157.2002.35601.x 12366723

[B66] Akman O Karson A Aker RG Ates N Onat FY Hippocampal kindling in rats with absence epilepsy resembles amygdaloid kindling Epilepsy Res 2008 81 211 219 10.1016/j.eplepsyres.2008.06.004 18657396

[B67] Akman O Karson A Aker RG Ates N Onat FY Perirhinal cortical kindling in rats with genetic absence epilepsy Neurosci Lett 2010 479 74 78 10.1016/j.neulet.2010.05.034 20560164

[B68] van der Staay FJ Schuurman T van Reenen CG Korte SM Emotional reactivity and cognitive performance in aversively motivated tasks: a comparison between four rat strains Behav Brain Funct 2009 5 50 10.1186/1744-9081-5-50 20003525 PMC2805679

[B69] Weiss SM Wadsworth G Fletcher A Dourish CT Utility of ethological analysis to overcome locomotor confounds in elevated maze models of anxiety Neurosci Biobehav Rev 1998 23 265 271 10.1016/S0149-7634(98)00027-X 9884119

