# Erratum: Age-dependent decline in learning and memory performances of WAG/Rij rat model of absence epilepsy

**DOI:** 10.1186/s12993-014-0052-6

**Published:** 2015-03-28

**Authors:** Ayşe Karson, Tijen Utkan, Fuat Balcı, Feyza Arıcıoğlu, Nurbay Ateş

**Affiliations:** Medical School, Department of Physiology, Kocaeli University, Umuttepe, Kocaeli 41380 Turkey; Medical School, Department of Pharmacology, Kocaeli University, Kocaeli, Turkey; Department of Psychology, Koç University, Istanbul, Turkey; Faculty of Pharmacy, Department of Pharmacology, Marmara University, Istanbul, Turkey

After publication of this work [1], the authors noticed that a different normalization factor was used for the graphical representation of “% time spent in correct quadrant” (Figure four C) (Figure 1). The correct figure and figure legend are included this erratum. Note that this does not change any of the statistical results or conclusions.

**Figure 1:**
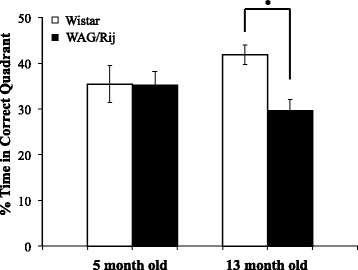
**Percentage of time spent in correct quadrant.** *Indicates p < .05.
